# Effect of omega-3 supplements or diets on fertility in women: A meta-analysis

**DOI:** 10.1016/j.heliyon.2024.e29324

**Published:** 2024-04-06

**Authors:** Shivtia Trop-Steinberg, Michael Gal, Yehudith Azar, Rachel Kilav-Levin, Eliyahu M. Heifetz

**Affiliations:** a Jerusalem College of Technology, Faculty of Life and Health Science, P.O.B. 16031, Jerusalem, Israel; b Hadassah Medical Center, Bone Marrow Transplantation Unit, P.O.B. 12000, Jerusalem, Israel; c Shaare Zedek Medical Center, IVF Unit, Department of Obstetrics and Gynecology, P.O.B. 3235, Jerusalem, Israel; d Israel and Hebrew University School of Medicine, Jerusalem, Israel

**Keywords:** Omega-3 fatty acids, Pregnancy rate, Fertilization rate, Systematic review, Meta-analysis

## Abstract

**Objective:**

This study aimed to assess the effect of increased omega-3 consumption on fertilization rates and the probability of women getting pregnant. This study is needed because different perspectives exist regarding the use of omega-3 fatty acids in enhancing fertility among women with reproductive issues, and information for those planning a spontaneous pregnancy is limited.

**Methods:**

PubMed, Clinical Trials, CINAHL/EBSCO, Medline Complete, Cochrane Library, and Google Scholar were searched for articles published until April 2021, and the search was limited to articles in English language. The search strategy included the following key words: “*in-vitro* fertilization (IVF),” “intracytoplasmic sperm injection techniques (ICSI),” “pregnancy,” “omega-3 fatty acid,” “alpha-linolenic acid,” “eicosapentaenoic acid,” “docosahexaenoic acid,” “n-3 polyunsaturated fatty acid,” and “fish oil and seafood.” Studies reporting female fertility occurring naturally or IVF/ICSI concurrent with omega-3 intake were included. Retrospective studies, studies including postmenopausal women, and unevenly matched control and study groups were excluded. To assess bias, we used the Cochrane Handbook for Systematic Reviews of Interventions, version 5.1.0. To synthesize the findings from the studies included in this review, a meta-analysis was conducted using calculated or extracted odds ratios (OR) of clinical pregnancies and fertilization rates for each group in each study.

**Results:**

We included six trials involving 1789 women who received fertility treatment, four trials involving 2607 women who conceived naturally, and three trials involving 1725 oocytes for fertility rates. Aggregated ORs for the effects of omega-3 on pregnancies were 1.74, 1.36, and 2.14 for women who received fertility treatment, those who conceived naturally, and fertilization rate, respectively. All these results were significant (*p* ≤ 0.01), although they had high heterogeneity I^2^>68 %.

**Conclusion:**

This systematic review and meta-analysis suggest that omega-3 intake significantly improves women's pregnancy and fertilization rates; however, the high heterogeneity in this review somewhat limits its interpretation. Therefore, further prospective randomized studies are necessary to better understand this relationship.

## Highlights

• Omega-3 intake enhances pregnancy rates among women undergoing ICSI/IVF.
• Omega-3 intake improves fertilization rates.
• Omega-3 enhances pregnancy outcomes in non-intervention conception.

## 1 Introduction

Worldwide, approximately 15 % of couples of reproductive age experience infertility [1,2]. Female-related causes of infertility include ovulatory disorders, poor egg quality, mechanical factors, endometriosis etc., which account for subfertility in 40–50 % of affected couples [[2], [3], [4]].

Omega-3 fatty acids, categorized as essential polyunsaturated fats that are vital for health, must be acquired from diet because the human body cannot synthesize them independently [5,6]. Alpha-linolenic acid (ALA), eicosapentaenoic acid (EPA), and docosahexaenoic acid (DHA) are obtained from plants (ALA), fish, and seafood sources (EPA and DHA). Individuals who do not consume fish can obtain ALA from plant-based sources, but the conversion of ALA to EPA and DHA in the body is not efficient [7,8].

Omega-3 fatty acids support fertility by improving hormonal balance, oocyte quality, embryo implantation [7], menstrual cycle function [8], and sperm health [5]. Additionally, they mitigate inflammation, which could interfere with proper function of reproductive organs [[5], [6], [7], [8]].

A diet rich in omega-3 polyunsaturated fatty acids (ώ3-PUFA) has been shown to improve female fertility in both animal and human studies [5,6]. Conversely, omega-3 deficiency and excessive omega-6 exposure are associated with increased risks of miscarriage and prematurity [[9], [10], [11]]. Prostaglandin synthesis and steroidogenesis modulated by omega-3 have been shown to improve uterine function, regulate hormonal secretion, and decrease the likelihood of developing endometriosis, thereby increasing fecundability [[9], [10], [11]]. Women on a diet containing high levels of omega-3 fatty acids had higher circulating levels of estradiol and improved embryo quality [7]. Women with polycystic ovary syndrome (PCOS) who were administered omega-3 supplementation over an eight-week study period showed decreased luteinizing hormone levels, lower luteinizing hormone/follicle stimulating hormone ratios, and lower concentrations of adiponectin [12]. Increased fecundability has been observed among North American women consuming a diet high in omega-3 [13].

Additional supporting evidence has been found in studies performed on other mammals, such as cows, that were fed omega-3 supplements; they were found to have larger follicles and increased fertility [11,13]. Although the diets of cows differ from those of humans, it is possible that their effects on the reproductive system are similar. These findings are not directly valid for humans, but are certainly a support, especially considering that not every experiment can be performed on humans due to ethical and other limitations.

However, conflicting results regarding the relationship between PUFA and female fertility have been reported [14,15]. Women with higher ratios of serum linoleic acid (LA) to ALA who underwent *in-vitro* fertilization (IVF) or embryo transfer had higher pregnancy rates compared to those with lower LA to ALA ratios [16]. Epigenetic modifications due to environmental exposure to specific dietary components at critical stages, including fertilization, gametogenesis, and/or early embryonic development, may have important effects on pregnancy and fetal growth and development [2,[17], [18], [19]].

The identification of dietary or other environmental factors that can be easily corrected may contribute to improving the fecundability and outcomes of assisted reproduction [20]. One of these factors could be omega-3, as it affects important and necessary parameters of fertility. However, opinions on the effect of omega-3 on getting pregnant in women treated for infertility differ. Additionally, information on the effect of omega-3 supplementation in women planning a spontaneous pregnancy are limited. Therefore, we assessed the effects of omega-3 fatty acid intake via supplementation or diet on fertility and pregnancy in women treated for infertility.

## 2 Methods

Study selection, assessment of eligibility criteria, data extraction, and statistical analysis were performed using a predefined protocol registered in PROSPERO (CRD42021216233). This review followed the Preferred Reporting Items for Systematic Reviews and Meta-Analyses (PRISMA) guidelines.

### 2.1 Information sources and search strategy

Two authors (TSS and KLR) searched for studies published in PubMed, ClinicalTrials, CINAHL/EBSCO, Medline Complete, Cochrane Library, and Google Scholar until April 2021, and limited to English language. Search terms/keywords were divided into two subsets. The first subset included the following terms: OR {“female fertility”, “infertility”, “pregnancy”, “Polycystic Ovary Syndrome (PCOS)”, “reproductive health”, “menstrual cycle”, “*in-vitro* fertilization (IVF)”, “intracytoplasmic sperm injection techniques (ICSIs)” [2,21] and “recurrent miscarriage”}. The second subset included the following terms: OR {“omega-3 fatty acid”, “ω-3 fatty acid”, “n-3 fatty acid”, “n-3 PUFA”, “α-linolenic acid (18:3n-3) ALA”, “eicosapentaenoic acid (20:5n-3) EPA”, “docosapentaenoic acid (22:5n-3) DPA”, “docosahexaenoic acid (22:6n-3) DHA”, “n-3 polyunsaturated fatty acid”, and “fish oil and seafood”}.

To ensure that the review search was as complete and current as possible, we performed additional searches for each source, as indicated above. Additionally, a snowball approach was employed, which involved a manual search of bibliographies from selected papers to identify additional relevant references.

### 2.2 Study selection and eligibility criteria

The criteria used to decide whether a study will be included in this review were as follows: 1) trials that assessed the effects of omega-3 fatty acids in the treatment of fertility (with no limits on the method of randomization, blinding, or language of publication); 2) women undergoing IVF treatment or other non-IVF treatments and those not undergoing any fertility treatment; and 3) if the interventional therapy is omega-3 fatty acid treatment, and control therapies include blanks, placebo, and lifestyle interventions.

Exclusion criteria includes studies involving postmenopausal women (aged >50 years).

Studies reporting female fertility occurring naturally or through IVF/ICSI, studies concurrent with omega-3 intake or omega-3-rich diet, and studies that met the inclusion criteria were included (Table A.1). However, not all the studies were relevant for the meta-analysis. Due to ovarian aging in postmenopausal women, studies involving postmenopausal women (or women aged >45 years) were excluded. Additionally, studies that tested omega-3 in erythrocytes [22] (reasoning for this follows below) and studies in which the control group was not evenly matched with the study group were excluded [23,24]. Furthermore, to determine the effect of omega-3 on pregnancy and fertilization, we selected only studies in which the distinguishing factor between study and control groups was the level of omega-3 administered. Therefore, studies that dealt with diet and did not clearly state the amount of omega-3/fish intake in each of the compared groups were excluded [25]. Studies that compared groups based on pregnancy success rates and retrospectively extracted information on omega-3 intake were excluded [26]. Additionally, there were also studies that included, in addition to omega-3, physical activity and various diets (Table A.1), most of which were rejected, with the exception of articles that compared different doses of omega-3 and their effects on fertility.

**Fig. 1:**
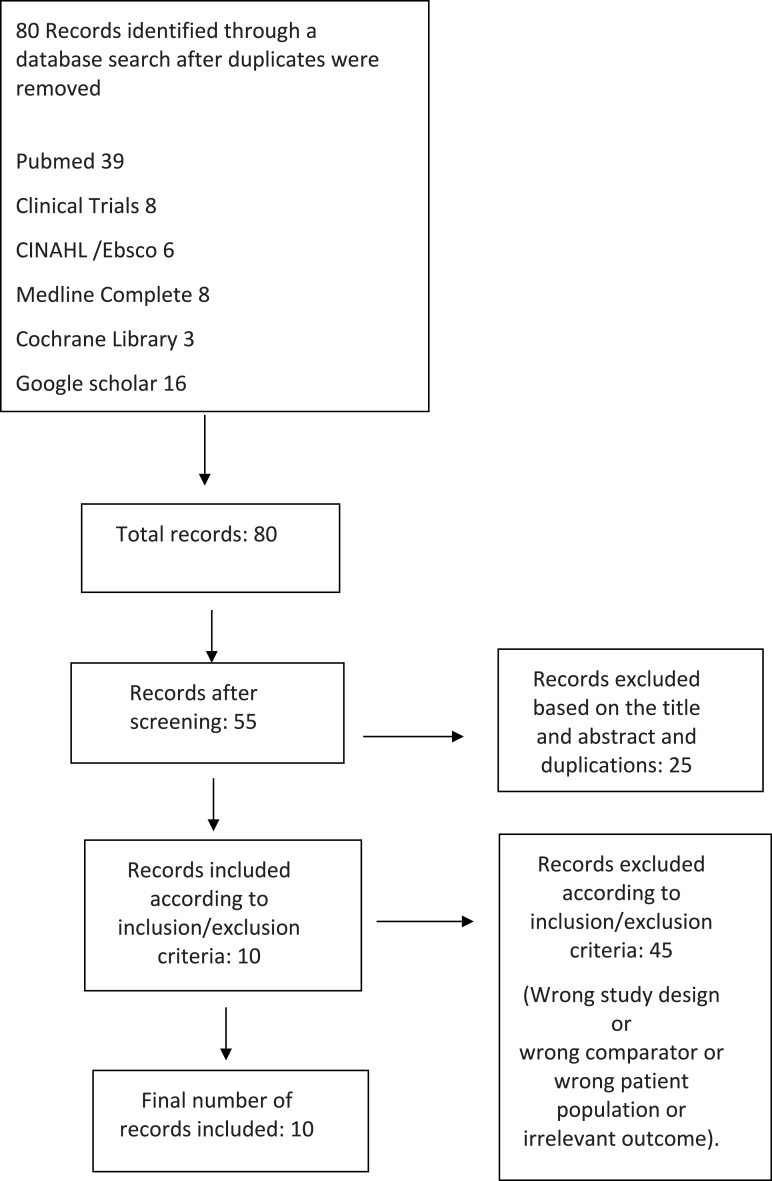
Flow diagram of the search article selection.

The initial search yielded 80 articles. Titles and abstracts were reviewed, and 25 articles that did not conform to the eligibility criteria were excluded. If the title or abstract appeared to meet the eligibility criteria or if it was insufficient to determine the eligibility of the study, the full texts of the articles were retrieved for further evaluation. Of the remaining 55 articles, 45 were excluded, leaving 10 studies for inclusion (Fig. 1). Contrary to our original plan, as outlined in the PROSPERO protocol, after conducting a literature search and assessing the number of randomized clinical trials (RCTs) available, we included all relevant studies, regardless of the study design.

### 2.3 Types of outcome measures

The primary outcome of interest in this study was pregnancy, which was defined in the broadest sense as the attainment of at least a positive result on a urine pregnancy test. The secondary outcome was fertilization, defined as the chance of obtaining a fertilized oocyte (versus the fertilization rate of a woman as a whole).

### 2.4 Data extraction

Two authors who reviewed the literature (TSS and KLR), selected studies that met the eligibility criteria, and independently extracted and tabulated the data using a standardized data extraction form. The following data were obtained: sample size, number of females in the intervention group, number of females in the control group, number of pregnancies in the intervention group, and number of pregnancies in the control group. The odds ratio (OR) of clinical pregnancies between groups for each study was extracted or calculated in cases where raw data were not available.

### 2.5 Data synthesis

The aggregated effect size (OR), its *p*-value, and 95 % confidence interval (CI) with lower and upper limits were computed by performing a meta-analysis. Additionally, the heterogeneity between studies in each meta-analysis was assessed using the *Q* statistic. The *Q* statistic is a chi-square statistic (with degrees of freedom equal to *k*–1) that reflects the variability among the effect estimates resulting from true heterogeneity rather than sampling errors. Based on the Q statistic, the I^2^ statistic, which is an intuitive and simple expression of the inconsistency of study results, was also extracted. Any value of I^2^ >50 % was considered highly inconsistent and required further consideration. A forest plot was presented for each meta-analysis to visualize the results. All of these were calculated using the “metafor” package for the statistical program R [27].

The primary outcomes were reported, and all outcome measures were pre-specified in the study protocol.

To calculate the pregnancy rate, data from six trials involving 1789 patients who underwent IVF or ICSI [[28], [29], [30], [31], [32], [33]] were used. Furthermore, to broaden the sample to include patients who conceived after receiving any kind of clinical treatment, the data were combined with those from a separate study [34] that included participants with PCOS who were treated with clomiphene citrate and received either omega-3 or a placebo. Moreover, data from three trials [13,35,36] involving 2607 patients who conceived naturally (did not undergo fertility treatment) and met the inclusion criteria were used.

The characteristics of pregnancy in fertility treatment and non-treatment groups are presented in Tables A.2 and A.3, respectively.

In the six studies that involved fertility treatment [[28], [29], [30], [31], [32], [33]], embryos transfer was performed in the cleavage stage, on day two or three after oocyte retrieval. In two of these six trials [28,29], the women were administered omega-3 supplements, whereas in the other four trials, the women followed a fish diet. Of the four trials involving patients who did not undergo fertility treatment [13,35,36], the women took omega-3 supplements in one study [35], whereas in the other three trials, the women followed fish diets. In all the studies, including diets (except for one [13]), the omega-3 component was specific to fish. We did not examine the groups according to the mode of omega-3 intake (i.e., supplement or fish diet), because, on the one hand, both methods resulted in an increase in omega-3 components in the diet, and on the other hand, there were not enough data to consider these as separate groups.

To calculate the fertilization rate, we used data from three trials involving 1725 oocytes retrieved from patients who underwent fertility treatment [29,32,37]. The fertility rate was determined by dividing the number of oocytes with two pronuclei, divided by the number of inseminated or injected metaphase II oocytes. In two of the three trials, the women took omega-3 supplements, whereas in one trial, they followed a fish-based diet. The characteristics of the studies included in the fertilization rate calculations are presented in Table A.4.

### 2.6 Assessment of risk of bias

To assess bias risks, two authors (KLR and STS) checked the risk of bias, according to the Cochrane Handbook for Systematic Reviews of Interventions version 5.1.0 [38] for the RCTs [29,37], and Newcastle-Ottawa Scale for the other studies [13,28,[30], [31], [32], [33],^35^,^36^]. For the RCTs, the biases assessed were selection bias (random sequence generation and allocation concealment), performance bias (blinding of participants and personnel), detection bias (blinding of outcome assessment), attrition bias (incomplete outcome data), reporting bias (selective outcome reporting), and other sources of bias. Discrepancies between the reviewers were resolved through discussions that included two additional authors (AY and GM) until a consensus was reached.

## 3 Results

### 3.1 Study selection

Data were included from 11 studies. To compare the aggregated OR effect of omega-3 intake (vs. non-intake) on clinical pregnancies in the treated groups, we used effect sizes from six studies [[28], [29], [30], [31], [32], [33]] involving 1789 women (Table A.5). To assess the aggregated OR effect of omega-3 intake (vs. non-intake) on clinical pregnancies in the groups not treated for conception, we used four different effect sizes from three studies [13,35,36] involving 2607 women (Table A.6).

To assess the aggregated OR effect of omega-3 intake (vs. non-intake) on fertilization rates, we used effect sizes from three studies [29,32,37] (two of which were also used in the treated group) involving 1725 oocytes (Table A.7).

### 3.2 Study characteristics

Tables A.2, A.3, and A.4 present the study characteristics. Since there were only a few RCTs, we finally included 11 studies, two RCTs [29,37] and nine cohort studies [13,28,[30], [31], [32], [33],^35^,^36^]. These trials were conducted in eight different countries from three continents, including America, Europe, and Asia. The causes of infertility remain largely unspecified in most studies; one study states that the participants had a “history of unexplained total fertilization failure” [37], and others just acknowledge those that were excluded. “Women with endometriosis and male infertility requiring operation were excluded” [30,31,33]. The mean of omega-3 intake varied among the studies, and are presented in Tables A.2–A.4.

In the group that underwent fertility treatment [[28], [29], [30], [31], [32], [33]], the duration of omega-3 intake ranged between three weeks to two months, and the duration of the fish-based diet was either 1–2 months or the entire previous year. In the group that did not undergo fertility treatment [13,35,36], omega-3 intake or a fish-based diet lasted between three months and one year. The consumption of omega-3 is described in Tables A.2–A.4, either in the form of capsules or through dietary intake, as assessed via questionnaires based on a high or low quartile ranking for omega-3 or fish consumption. The results did not reveal a difference between studies with varying durations of food intake or diet.

Additionally, the age range of the participants who took omega-3 (treated: 20–41-years-old; non-treated: 18–44-years old) did not affect the direction of the outcome.

### 3.3 Risk of bias

Complete agreement was reached between the two authors for both assessments. Some studies followed unclear randomization procedures and were rated as having an unclear risk of bias. Other studies employed adequate methods of random sequence generation, which we rated as having a low risk of bias. We rated studies reporting that their omega-3 and placebo/control groups were similar in shape and size, or those that were double-blinded as having a low risk of blinding bias. However, we rated all the trials that included a diet, and hence used a history questionnaire method and were not blinded, as having a high risk of bias. Most trials reported relevant data and fully reported their outcomes; therefore, we rated them as having a low risk of bias. Studies that included a diet rather than an omega-3 supplement were also rated as having a high risk of bias, as well as other biases, such as studies that examined the effect of omega-3 serum levels on pregnancy. Graphical summaries of bias risk assessment are presented in Table A.8 and Figure A.1–4. The results for each outcome are presented in separate sections below.

## 4 Synthesis of results

### 4.1 Clinical pregnancy in treated groups

**Fig. 2:**
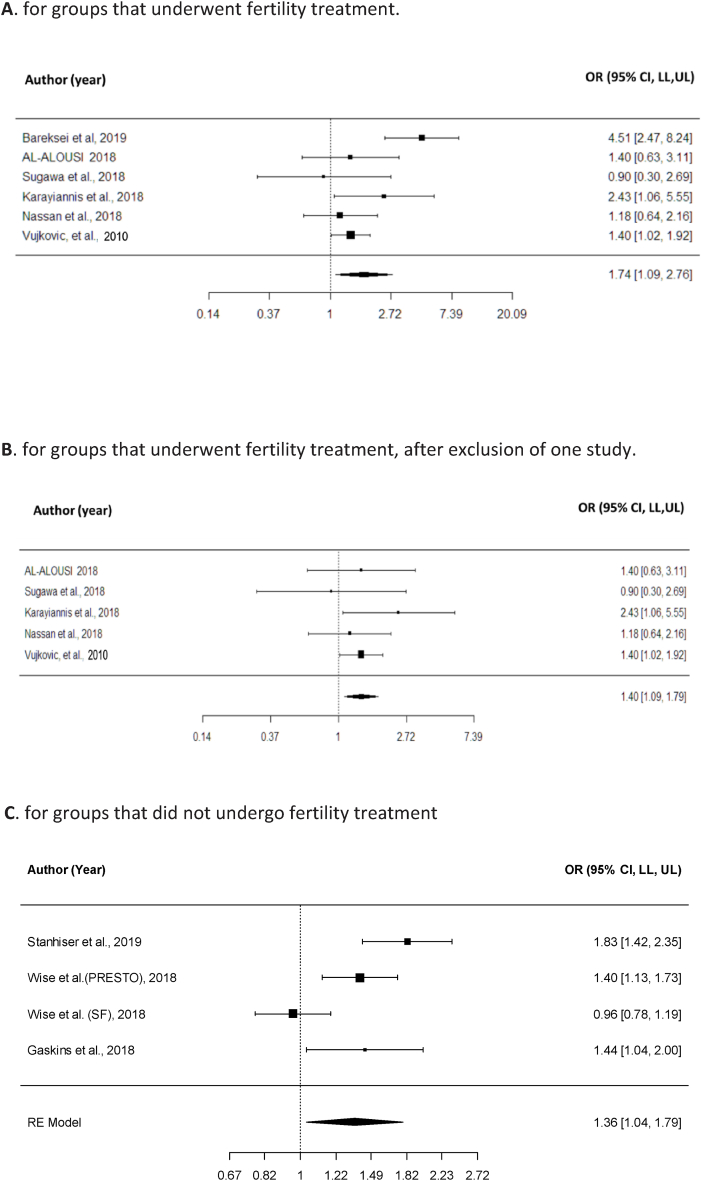
Forest plot of OR effect of omega-3 intake (intervention:control) on pregnancy rates: **A**. for groups that underwent fertility treatment. **B**. for groups that underwent fertility treatment, after exclusion of one study. **C**. for groups that did not undergo fertility treatment. The line for each study represents a 95 % confidence interval (lower limit and upper limit) for the effect (the numbers are found on the right side of the figure). The reference line of the OR is 1, which represents a null effect. The aggregated effect size is represented in the lower portion of the figure. Forest plot showing the aggregated effect size: **A**. For groups who underwent fertility treatment. **B**. For groups who underwent fertility treatment, after exclusion of one study. **C**. For groups without fertility treatment.

To compare the aggregated OR effect of omega-3 intake on clinical pregnancies in the treated groups, we used effect sizes from six studies [[28], [29], [30], [31], [32], [33]] (Table A.5). The results from the random effects model showed that the aggregated effect size of omega-3 to achieve clinical pregnancy was *OR* = 1.74 (95 % CI [1.09, 2.76]). This result was significant (*z* = 2.32, *p* = 0.02) (Fig. 2A), suggesting that omega-3 increases pregnancy rates in treated women.

However, a significantly high heterogeneity was found between the studies (*I*^*2*^ = 68.24 %, *Q* [5] = 15.10, *p* = 0.01). This high heterogeneity was probably due to the effect size reported by Bareksei et al. (2019) [28], with an OR of 4.51, which is substantially larger than the effects in other studies.

Therefore, we performed a sensitivity analysis in which this study was excluded. Results without Bareksei et al. (2019) [28] were as follows: *OR* = 1.40 (95 % CI [1.09, 1.79]). This result was significant (*z* = 2.67, *p* < 0.001) (Fig. 2B). In this analysis, the heterogeneity between studies was extremely low and nonsignificant (*I*^*2*^ = 0 %, *Q* [4] = 2.64, *p* = *0.*61).

It is important to note that although Karayiannis (2018) [31] referred to patients who were treated for the first time, that is, without a previous IVF attempt or pregnancy, his results were in line with those of all other studies and hence did not add to the heterogeneity.

Additionally, it should be noted that in all cases involving women who underwent treatment, the OR was >1, except for that in the study by Sugawa et al. (2018) [30], in which the OR was <1.

In addition to the six studies on women who underwent fertility treatment, we also included findings from a study [34] that compared the pregnancy rates of women with PCOS who underwent ovulation induction using clomiphene citrate and received either an omega-3 supplement or a placebo. The result, *OR* = 1.80 (95 % CI [1.16, 2.77]), was significant (*z* = 2.63, *p* < 0.001); however, a significant heterogeneity was found between the studies (*I*^*2*^ = 62.95 %, *Q* [6] = 15.63, *p* = 0.01).

### 4.2 Clinical pregnancy in untreated groups

To assess the aggregated OR effect of omega-3 intake (vs. non-intake) on clinical pregnancies in the groups not treated for conception, we used four different effect sizes from three studies [13,35,36] (since one of the articles compared two distinct populations from different continents [14]; Table A.6) involving women who conceived naturally (i.e., without fertility treatment). The results showed that the aggregated effect size of using omega-3 diet to achieve clinical pregnancy without treatment was OR = 1.36 (95 % CI [1.04, 1.79]). This result was significant (*z* = 2.23, *p* = 0.03) (Fig. 2C), suggesting that omega-3 increases pregnancy rates in untreated women. However, a significantly high heterogeneity was found between the studies (*I*^*2*^ = 79.56 %, *Q* [2] = 15.76, *p* = 0.001). This heterogeneity was probably due to the effect size reported by Wise et al. specifically the Snart Fraeldre (SF) cohort (2018) [13], with an OR of 0.96 (Table A.6), which is considerably lower than the effects in other studies.

Two possible sources of high heterogeneity were found in this analysis: 1) geographic location (USA vs. Europe) leads to variations in dietary practices influenced by cultural norms or the availability of different types of foods (e.g., wild-caught vs. farmed fish [39]; and 2) mode of intake (omega-3 vs. fish diet), which may influence the body's absorption levels. Therefore, two sensitivity analysis were conducted: 1) excluding the study by Wise et al. SF (2018) [13], which means including only USA studies, this resulted in a non-significant low heterogeneity (I^2^ = 30.66 %, *p* = 0.26) and significant OR = 1.54 (1.29–1.85; *p* < 0.0001); 2) after excluding the study by Stanhiser et al. (2019) [34], which means including only fish diet, there was even a higher heterogeneity (I^2^ = 87.38 %, p = 0.0004) and non-significant OR of 1.34 (0.93–1.94; *p* < 0.11).

### 4.3 Effect of omega-3 on fertilization rates

**Fig. 3:**
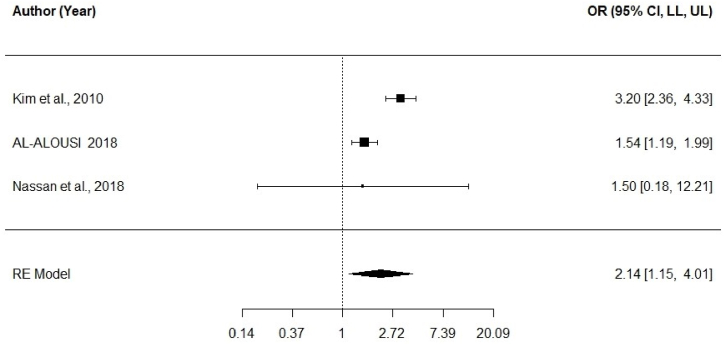
Forest plot of OR effect of omega-3 (intervention:control) on fertilization rates. The line for each study represents a 95 % confidence interval (lower limit and upper limit) for the effect (the numbers are found on the right side of the figure). The reference line of the OR is 1, which represents a null effect. The aggregated effect size is represented in the lower portion of the figure.

To assess the aggregated OR effect of omega-3 intake (vs. no intake) on fertilization rates, we used effect sizes from three studies [29,32,37] (Table A.7). In their analysis, the study by Karyiannis et al. (2018) [31] was not included because the data in this study were presented as median and not as mean, as in the other three studies that were included. The results showed that the aggregate effect size of omega-3 on the fertilization rate was *OR* = 2.14 (95 % CI [1.15, 4.01]). This result was significant (*z* = 2.38, *p* = 00.0017) (Fig. 3). Significant heterogeneity was found among the studies (*I*^*2*^ = 83.42 %, *Q* [2] = 13.13, *p* = 0.001). These results suggest that omega-3 supplementation enhances fertility rates.

This high heterogeneity was likely due to the effect size reported by Kim et al. (2010) [37], with an OR of 3.20, which was substantially larger. However, given that the final result was significant, even without this study, we decided to leave it in the report.

### 4.4 Omega-3 serum levels

Five studies examined the effects of omega-3 serum levels on pregnancy rates. Chiu et al. (2018) [20] tested the serum of women who underwent fertility treatment. They showed that the higher the omega-3 level, the greater the chance of pregnancy. Stanhiser et al. (2020) [40] and Mirabi et al., 2017 [41] conducted a retrospective study in which they compared the omega-3 serum levels of two groups of women who tried to conceive spontaneously: one group included women who succeeded in conceiving, and the other did not. Stanhiser et al. (2020) [40] did not find any significant differences between the two groups, whereas Mirabi et al. (2017) [41] found that omega-3-EPA levels in the pregnant group were higher. Jungheim et al. (2013) [16] examined the ratio of LA to ALA, and observed that those with a higher ratio were more likely to conceive.

In contrast, Mumford et al. (2018) [42] conducted a baseline study in which they tested omega-3 levels prior to pregnancy. They evaluated the time to pregnancy and number of menstrual cycles to achieve pregnancy.

These studies were not part of the meta-analysis because there was no uniformity in the way they were performed. This is with regards to testing omega-3 serum levels, or the inclusion of groups that underwent fertility treatments as opposed to those without fertility treatments, or the stage in which omega-3 was tested. Therefore, it is irrelevant to include all of these in the same analysis.

## 5 Discussion

In this systematic review, a wide range of studies were incorporated to examine the potential of omega-3 fatty acids to enhance fertility in women. These studies included variations in the duration of omega-3 consumption, mode of intake (pill or dietary source), diet type, reasons for infertility, and research methodologies. Despite the substantial heterogeneity observed across these studies, a decision was made to include them collectively, as they represent existing information in the current literature. It is worth mentioning that in certain instances, this heterogeneity did not yield significant differences, as elaborated in the results and discussed later.

The uniqueness of this study is that it mainly focused on the likelihood of achieving pregnancy. It focuses on the ultimate outcome that concerns women actively planning for pregnancy, either through medical intervention or spontaneous conception. The key measure of interest is the definitive occurrence of pregnancy, considering that not every successful fertilization culminates in a clinical pregnancy.

### 5.1 Principal findings

Our findings suggest that omega-3 fatty acid intake may improve pregnancy and fertilization rates, both for women undergoing treatment to conceive and for those who do not use any intervention. Although there are limited studies on this subject, we observed that, overall, those we reviewed revealed a similar pattern. This implies that omega-3 fatty acid intake, whether through supplements or fish consumption, is associated with improved pregnancy and fertilization rates in women aged 20–44 years. This is reflected in the fact that in all studies of women who underwent treatment for pregnancy, the chance of becoming pregnant after receiving omega-3 fatty acids was higher than that in a control group. The exception was a study by Sugawa et al. (2018) [30], in which a group that received omega-3 did not show an advantage over a control group. This may be because the sample size used in the study by Sugawa et al. (2018) [30] was relatively small (35 % smaller than the previously mentioned study), especially when it comes to a study that is considered weak because it was conducted through a questionnaire, where a larger sample is needed. Simultaneously, the different origins of women in this study may have influenced these unusual results. However, the fertilization method (ICSI or IVF) should be considered separately in future studies because it introduces diversity.

Moreover, serum reports indicated that among women who underwent fertility treatment, those who became pregnant had higher omega-3 levels compared to those who did not. Notably, there have been no studies involving long-term data on the efficacy of omega-3 fatty acids in women. The study duration was from three weeks to 12 months. The effects of omega-3 fatty acids have been observed after two or three months [43].

### 5.2 Comparison with existing literature

Sunderam et al. (2019) [44] conducted a meta-analysis to evaluate the difference in fertilization rates between ICSI and IVF, without examining any relationship with omega-3. They showed that fertilization rates did not differ between conventional IVF and those resulting from ICSI in patients aged 38 years with non-male factor infertility. In contrast, when examining the data of the studies included in this review on women undergoing treatment to conceive, we found that the effect size reported by Bareksei et al. (2019) [28] (in which only IVF was used) was substantially higher than that in other studies (in which either ICSI or IVF was used). This suggests that IVF results in higher fertilization rates. A conjecture that might explain this is that the outlier study did not report the age or body mass index of the participants; therefore, it might be that these factors were different from the values reported in other studies. Additionally, Bareksei et al. (2019) [28] had a larger sample size than other studies, which could have resulted in a higher statistical power to gain a more significant result than the other studies.

Nevertheless, we conducted a sensitivity analysis excluding the study by Bareksei et al. (2019) [28], which eliminated this heterogeneity. However, the outcome indicated a similar tendency, namely a significant OR of >1; therefore, we left it in the analysis. Nonetheless, it appears that the mode of fertilization is a source of heterogeneity, and should not be considered as one group in future studies. Additionally, the heterogeneity found in this meta-analysis was not attributable to the mode of omega-3 intake (omega-3 vs. fish diet). Relative homogeneity was found between studies that used different intake modes. Therefore, according to our results, the form of omega-3 diet intake did not affect the omega-3 effect size reported in other studies. The outcomes of women with PCOS who underwent ovulation induction using clomiphene citrate without IVF treatment showed the same tendency [34].

Except for Nassan et al. (2018) [32], a study that involved both ICSI and conventional insemination procedures, all other studies that examined the effect of omega-3 on fertilization rates included only patients who underwent ICSI. This difference did not affect the outcomes. Only the effect size reported by Kim et al. (2010) [37], which was larger than the others, influenced the heterogeneity of the outcome, although this was a prospective, randomized controlled study. These limitations may be explained by the different study sizes or variations in laboratory techniques between clinics and countries.

The most recent meta-analysis on this topic was conducted by Gatti et al. (2021) [45]. They evaluated whether increased dietary intake of PUFAs or monounsaturated fatty acids during the periconception period had beneficial effects on achieving pregnancy; thus, their study included the effects of omega-3 on pregnancy rates. In contrast to the current findings, their conclusion was that omega-3 intake did not have a significant effect on pregnancy rates. However, there were several design differences between the two meta-analytic studies: (a) unlike our study, they did not distinguish between patients who underwent fertility treatments and those who did not [45]; (b) in contrast to our analysis, which excluded studies that measured omega-3 levels in serum, their studies overlooked the difference in the source of omega-3 measurements [20,42]. The reason for this exclusion was the absence of an evidence directly linking prolonged omega-3 intake to serum omega-3 levels in humans. It has been shown that prolonged omega-3 *intake* prevented shortening of pregnancy duration [46]. However, serum levels of omega-3 were not directly correlated with pregnancy duration [46]; and (c) in one of the studies included in the other meta-analysis, the study group had olive oil intake in addition to omega-3 intake; however, the control group did not have olive oil [23], which might have a positive effect on achieving pregnancy [45].

Although the serum results are not consistent, they suggest that in women who underwent fertility treatments, omega-3 levels are higher in those who became pregnant than in those who did not.

As previously mentioned, the likelihood of achieving pregnancy, in women with PCOS, was higher (34). Nevertheless, studies indicate that ALA, in conjunction with the natural polyol myo-Inositol (myo-Ins) and its isomers (D-Chiro-Inositol and D-Chiro-Ins), which act by associating with ALA, could be advantageous in the treatment of PCOS by improving insulin resistance [47]. The American Heart Association recommends the consumption of at least two meals containing fatty fish per week to ensure adequate intake of EPA and DHA [48]. Additionally, vitamin D has been suggested to play a beneficial role in addressing insulin resistance and enhancing endometrial receptivity [49].

### 5.3 Strengths and limitations

The strengths of our meta-analysis are that the studies were based solely on the effects of omega-3 intake (via supplementation or an enriched diet). Additionally, we excluded studies in which omega-3 intake was based on serum tests (see the rationale of the study in the Discussion section) and studies with unevenly matched controls and study groups.

Furthermore, patients who underwent assisted reproductive treatments (i.e., IVF/ICSI) were differentiated from those who achieved spontaneous pregnancies. Moreover, this is one of the first meta-analyses to review the effects of omega-3 supplementation in patients whose omega-3 intake began prior to (natural or assisted) fertilization and pregnancy.

This meta-analysis has several limitations, including the limited availability of appropriate studies, particularly those focusing on pregnancy outcomes in individuals who did not undergo fertility treatment. Additionally, the lack of uniformity in how omega-3 is obtained and limited knowledge regarding the precise levels of omega-3 acquired in certain diets did not enable the determination of safe dose ranges, recommended doses, and optimal duration of use. Furthermore, it was not possible to separate the groups according to the type of omega fatty acid administered and mode and duration of intake because there were not enough studies to divide them into subgroups. Therefore, we added the results of all the groups. Although we observed a similar trend between the different methods of consuming omega fatty acid, it would be worthwhile to conduct studies that would examine the changes between the different treatment groups. Apart from attrition bias, there was a high risk of bias in all or most cases. Regarding heterogeneity, there was rather large variability in the type of control groups, mode of conception, and different countries, which is discussed further in the following sections.

## 6 Conclusions and implications

Our systematic review and meta-analysis suggested that omega-3 fatty acid intake through supplements or a fish-rich diet, may provide a novel benefit for women who want to conceive, because it is natural and nonintrusive. However, there is currently insufficient evidence to confirm this. Considering the limited information and its quality, we are unable to issue definitive recommendations regarding dosage and duration; however, we only provide a general recommendation for women trying to become pregnant to increase the amount of omega-3 in their diet through food supplements or fish. Therefore, additional randomized, double-blind clinical trials with larger sample sizes and longer durations are needed. Additionally, the experimental group should all receive the same form and dosage of omega-3 fatty acid or diet, whereas the control group should receive identical alternatives.

## Data availability statement

The data pertaining to this study have not been deposited in a publicly accessible repository, given that all relevant data are thoroughly detailed in the article, supplementary materials, or appropriately cited in the manuscript.

## CRediT authorship contribution statement

**Shivtia Trop-Steinberg:** Writing – review & editing, Writing – original draft, Supervision, Formal analysis, Data curation, Conceptualization. **Michael Gal:** Writing – review & editing, Formal analysis. **Yehudith Azar:** Writing – review & editing, Methodology, Formal analysis. **Rachel Kilav-Levin:** Writing – review & editing, Data curation. **Eliyahu M. Heifetz:** Writing – review & editing, Writing – original draft, Supervision, Methodology, Formal analysis, Conceptualization.

## Declaration of competing interest

The authors declare that they have no known competing financial interests or personal relationships that could have appeared to influence the work reported in this paper.

## Abbreviations

Alpha-linolenic acid: (ALA)
Docosahexaenoic acid: (DHA)
Eicosapentaenoic acid: (EPA)
Intracytoplasmic sperm injection techniques: (ICSIs)
*In-vitro* fertilization: (IVF)
Odds Ratio: (OR)
Omega-3 polyunsaturated fatty acids: (ώ3-PUFA)
Polycystic ovary syndrome: (PCOS)

## References

[1] Liang S., Chen Y., Wang Q., Chen H., Cui C., Xu X. (2021). Prevalence and associated factors of infertility among 20–49 year old women in Henan Province, China. Reprod. Health.

[2] Thoma M.E., McLain A.C., Louis J.F., King R.B., Trumble A.C., Sundaram R. (2013). Prevalence of infertility in the United States as estimated by the current duration approach and a traditional constructed approach. Fertil. Steril..

[3] Showell M.G., Brown J., Clarke J., Hart R.J. (2013). Antioxidants for female subfertility. Cochrane Database Syst. Rev..

[4] Showell M.G., Mackenzie-Proctor R., Jordan V., Hart R.J. (2017). Antioxidants for female subfertility. Cochrane Database Syst. Rev..

[5] Lass A., Belluzzi A. (2019). Omega-3 polyunsaturated fatty acids and IVF treatment. Reprod. Biomed. Online.

[6] Zarezadeh R., Mehdizadeh A., Leroy J., Nouri M., Fayezi S., Darabi M. (2019). Action mechanisms of n-3 polyunsaturated fatty acids on the oocyte maturation and developmental competence: potential advantages and disadvantages. J. Cell. Physiol..

[7] Hammiche F., Vujkovic M., Wijburg W., de Vries J.H., Macklon N.S., Laven J.S. (2011). Increased preconception omega-3 polyunsaturated fatty acid intake improves embryo morphology. Fertil. Steril..

[8] Nadjarzadeh A., Dehghani Firouzabadi R., Vaziri N., Daneshbodi H., Lotfi M.H., Mozaffari-Khosravi H. (2013). The effect of omega-3 supplementation on androgen profile and menstrual status in women with polycystic ovary syndrome: a randomized clinical trial. Iran. J. Reproductive Med..

[9] Calder P.C., Yaqoob P. (2009). Understanding omega-3 polyunsaturated fatty acids. Postgrad. Med..

[10] Safarinejad M.R., Hosseini S.Y., Dadkhah F., Asgari M.A. (2010). Relationship of omega-3 and omega-6 fatty acids with semen characteristics, and anti-oxidant status of seminal plasma: a comparison between fertile and infertile men. Clin. Nutr..

[11] Wathes D.C., Abayasekara D.R., Aitken R.J. (2007). Polyunsaturated fatty acids in male and female reproduction. Biol. Reprod..

[12] Nadjarzadeh A., Dehghani-Firouzabadi R., Daneshbodi H., Lotfi M.H., Vaziri N., Mozaffari-Khosravi H. (2015). Effect of omega-3 supplementation on visfatin, adiponectin, and anthropometric indices in women with polycystic ovarian syndrome. J. Reproduction Infertil..

[13] Wise L.A., Wesselink A.K., Tucker K.L., Saklani S., Mikkelsen E.M., Cueto H. (2018). Dietary fat intake and fecundability in 2 preconception cohort studies. Am. J. Epidemiol..

[14] Simopoulos A.P. (2010). Genetic variants in the metabolism of omega-6 and omega-3 fatty acids: their role in the determination of nutritional requirements and chronic disease risk. Exp. Biol. Med..

[15] Corella D., Ordovas J.M. (2012). Interactions between dietary n-3 fatty acids and genetic variants and risk of disease. Br. J. Nutr..

[16] Jungheim E.S., Frolova A.I., Jiang H., Riley J.K. (2013). Relationship between serum polyunsaturated fatty acids and pregnancy in women undergoing *in vitro* fertilization. J. Clin. Endocrinol. Metabol..

[17] Malireddy S., Kotha S.R., Secor J.D., Gurney T.O., Abbott J.L., Maulik G. (2012). Phytochemical antioxidants modulate mammalian cellular epigenome: implications in health and disease. Antioxidants Redox Signal..

[18] Luo L.L., Huang J., Fu Y.C., Xu J.J., Qian Y.S. (2008). Effects of tea polyphenols on ovarian development in rats. J. Endocrinol. Invest..

[19] Liu M., Yin Y., Ye X., Zeng M., Zhao Q., Keefe D.L. (2013). Resveratrol protects against age-associated infertility in mice. Hum. Reprod. (Oxf.).

[20] Chiu Y.H., Karmon A.E., Gaskins A.J., Arvizu M., Williams P.L., Souter I. (2018). Serum omega-3 fatty acids and treatment outcomes among women undergoing assisted reproduction. Hum. Reprod. (Oxf.).

[21] Nardelli A.A., Stafinski T., Motan T., Klein K., Menon D. (2014). Assisted reproductive technologies (ARTs): evaluation of evidence to support public policy development. Reprod. Health.

[22] Eskew A.M., Wormer K.C., Matthews M.L., Norton H.J., Papadakis M.A., Hurst B.S. (2017). The association between fatty acid index and *in vitro* fertilization outcomes. J. Assist. Reprod. Genet..

[23] Kermack A.J., Lowen P., Wellstead S.J., Fisk H.L., Montag M., Cheong Y. (2020). Effect of a 6-week “Mediterranean” dietary intervention on *in vitro* human embryo development: the Preconception Dietary Supplements in Assisted Reproduction double-blinded randomized controlled trial. Fertil. Steril..

[24] Nouri K., Walch K., Weghofer A., Imhof M., Egarter C., Ott J. (2017). The impact of a standardized oral multinutrient supplementation on embryo quality in *in vitro* fertilization/intracytoplasmic sperm injection: a prospective randomized trial. Gynecol. Obstet. Invest..

[25] Sun H., Lin Y., Lin D., Zou C., Zou X., Fu L. (2019). Mediterranean diet improves embryo yield in IVF: a prospective cohort study. Reprod. Biol. Endocrinol. : RB Elektron..

[26] Moran L.J., Tsagareli V., Noakes M., Norman R. (2016). Altered preconception fatty acid intake is associated with improved pregnancy rates in overweight and obese women undertaking in vitro fertilisation. Nutrients.

[27] Viechtbauer W. (2010). Conducting meta-analyses in R with the metafor package. J. Stat. Software.

[28] Bareksei A., Hafner G., Pfeiffer S., Schlatterer K. (2019). Effects of periconceptual omega-3-fatty acid supplementation on in vitro fertilization success and miscarriage rates in patients of a German fertility centre. Int. J. Clin. Exp. Med. Sci..

[29] Al-Alousi T.A., Ahmed Aziz A., Ali Al-Allak M., Sh, Al Ghazali B. (2018). The effect of omega-3 on the number of retrieved ova, fertilization rate, and embryonic grading in subfertile females experiences intracytoplasmic sperm injection management protocols. Biomed. Pharmacol. J..

[30] Sugawa M., Okubo H., Sasaki S., Nakagawa Y., Kobayashi T., Kato K. (2018). Lack of a meaningful association between dietary patterns and *in vitro* fertilization outcome among Japanese women. Reprod. Med. Biol..

[31] Karayiannis D., Kontogianni M.D., Mendorou C., Mastrominas M., Yiannakouris N. (2018). Adherence to the Mediterranean diet and IVF success rate among non-obese women attempting fertility. Hum. Reprod. (Oxf.).

[32] Nassan F.L., Chiu Y.-H., Vanegas J.C., Gaskins A.J., Williams P.L., Ford J.B. (2018). Intake of protein-rich foods in relation to outcomes of infertility treatment with assisted reproductive technologies. Am. J. Clin. Nutr..

[33] Vujkovic M., de Vries J.H., Lindemans J., Macklon N.S., van der Spek P.J., Steegers E.A. (2010). The preconception Mediterranean dietary pattern in couples undergoing *in vitro* fertilization/intracytoplasmic sperm injection treatment increases the chance of pregnancy. Fertil. Steril..

[34] Trop-Steinberg S., Heifetz E.M., Azar Y., Kafka I., Weintraub A., Gal M. (2023). Omega-3 intake improves clinical pregnancy rate in polycystic ovary syndrome patients: a double-blind, randomized study. Isr. Med. Assoc. J. : Isr. Med. Assoc. J..

[35] Stanhiser J., Jukic A.M., Steiner A.Z. (2019). Omega-3 fatty acid supplementation and fecundability. Fertil. Steril..

[36] Gaskins A.J., Sundaram R., Buck Louis G.M., Chavarro J.E. (2018). Seafood intake, sexual activity, and time to pregnancy. J. Clin. Endocrinol. Metabol..

[37] Kim C.-H., Yoon J.-W., Ahn J.-W., Kang H.-J., Lee J.-W., Kang B.-M. (2010). The effect of supplementation with omega-3-polyunsaturated fatty acids in intracytoplasmic sperm injection cycles for infertile patients with a history of unexplained total fertilization failure. Fertil. Steril..

[38] Higgins J.P., Thomas J., Chandler J., Cumpston M., Li T., Page M.J. (2019).

[39] Lundebye A.K., Lock E.J., Rasinger J.D., Nøstbakken O.J., Hannisdal R., Karlsbakk E. (2017). Lower levels of Persistent Organic Pollutants, metals and the marine omega 3-fatty acid DHA in farmed compared to wild Atlantic salmon (Salmo salar). Environ. Res..

[40] Stanhiser J., Jukic A.M.Z., Steiner A.Z. (2020). Serum omega-3 and omega-6 fatty acid concentrations and natural fertility. Hum. Reprod. (Oxf.).

[41] Mirabi P., Chaichi M.J., Esmaeilzadeh S., Ali Jorsaraei S.G., Bijani A., Ehsani M. (2017). The role of fatty acids on ICSI outcomes: a prospective cohort study. Lipids Health Dis..

[42] Mumford S.L., Browne R.W., Kim K., Nichols C., Wilcox B., Silver R.M. (2018). Preconception plasma phospholipid fatty acids and fecundability. J. Clin. Endocrinol. Metabol..

[43] Kesavulu M.M., Kameswararao B., Apparao C., Kumar E.G., Harinarayan C.V. (2002). Effect of omega-3 fatty acids on lipid peroxidation and antioxidant enzyme status in type 2 diabetic patients. Diabetes Metabol..

[44] Sunderam S., Boulet S.L., Kawwass J.F., Kissin D.M. (2020). Comparing fertilization rates from intracytoplasmic sperm injection to conventional *in vitro* fertilization among women of advanced age with non-male factor infertility: a meta-analysis. Fertil. Steril..

[45] Gatti C.R., Gomez Ribot D., Mariani J., Jawerbaum A. (2021). Unsaturated fatty acid intake during periconception and incidence of achieving pregnancy: a systematic review and meta-analysis. Front. Physiol..

[46] von Schacky C. (2020). Omega-3 fatty acids in pregnancy-the case for a target omega-3 index. Nutrients.

[47] Laganà A.S., Monti N., Fedeli V., Gullo G., Bizzarri M. (2022). Does Alpha-lipoic acid improve effects on polycystic ovary syndrome?. Eur. Rev. Med. Pharmacol. Sci..

[48] Jain A.P., Aggarwal K.K., Zhang P.Y. (2015). Omega-3 fatty acids and cardiovascular disease. Eur. Rev. Med. Pharmacol. Sci..

[49] Menichini D., Forte G., Orrù B., Gullo G., Unfer V., Facchinetti F. (2022). The role of vitamin D in metabolic and reproductive disturbances of polycystic ovary syndrome: a narrative mini-review. Int. J. Vitam. Nutr. Res..

