# Supplementary material for: Effect of omega-3 supplements or diets on fertility in women: A meta-analysis
Source: Heliyon. 2024 Apr 6;10(8):e29324. doi: 10.1016/j.heliyon.2024.e29324 (PMC11019195; doi:10.1016/j.heliyon.2024.e29324)
Supplement: Multimedia component 1 [file mmc1.docx]

##### Table A.1: Inclusion criteria

| P (Participants) | Women attending a reproductive clinic; women who underwent fertility treatment (mostly ICSI), and women who conceived naturally (without any treatment) |
| --- | --- |
| I (Intervention) | Omega-3 fatty acid administration with no limits regarding administration route, frequency or treatment dosage. The intervention can be omega-3 fatty acids only, omega-3 fatty acids plus Western medicine, or omega-3 fatty acids plus lifestyle interventions or exercise, or omega-3 fatty acids plus fish/seafood oil or seafood |
| C (Comparisons) | The control included blanks, placebo, lifestyle interventions, exercise, and diet with the lowest pattern of omega-3 |
| O (Outcomes) | Primary: Clinical pregnancy  Secondary: Fertilization rate |

##### Table A.2: Studies included in the calculation of the odds ratio effect of omega-3 intake on pregnancy rates among patients who underwent fertility treatment: Features and patient characteristics

| **Study** | **Study design** | **Total (N)** | **Patient characteristics** | **Country** | **Mean age,**  **y/ range** | **Mean BMI/ range** | **Type of Omega-3** | **Type of Control** | **Duration** | **Adjustment** |
| --- | --- | --- | --- | --- | --- | --- | --- | --- | --- | --- |
| Bareksei et al, 2019 [28] | Non-randomized | 1161 | IVF patients. History of at least 2 y miscarriages or unsuccessful IVF approaches | German | Not recorded | Not recorded | Omega-3 supplements (300 mg EPA + 200 mg DHA/day) | Group that did not take omega-3 supplements (cohort was generated from the Fertility Centre´s patient database**)** | For at least 3 weeks | NR |
| AL-Alousi 2018 [29] | Randomized, double-blind, placebo-controlled clinical trial | 115 | Subfertile ICSI patients.  No medical disorders or history of any diet in the previous three months. No tobacco or alcohol consumption, or supplementation of n-3 PUFAs in the past three months | Iraq | 20-40 | 18-34.99 | One 1000 mg omega-3 capsule daily | one daily placebo of 500 mg liquid paraffin | 8 weeks | NR |
| Sugawa et al., 2018 [30] | Diet history questionnaires | 70 | ICSI or conventional IVF patients.  Excluded were women with endometriosis and male infertility requiring operation | Japan | 36.7 | Not recorded | “Vegetable and seafood" dietary pattern highest (Q4) quartiles | “Vegetable and seafood" dietary pattern lowest (Q1) quartiles | During the month prior to egg retrieval | Age, BMI, parity, education, smoking experience, alcohol consumption and folate supplement use  Energy adjusted food g/1000 kcal were extracted by factor analysis |
| Karayiannis et al., 2018 [31] | diet history questionnaires | 165 | ICSI, using their own oocytes, without a previous IVF attempt or pregnancy, do not have endometriosis, or a history of disorders like diabetes, etc., did not change their dietary habits for at least the previous 6 months | Greece | 22-41 | < 30 | highest tertial (model 3) which include fish | lowest tertial (model 1) | During the month prior to egg retrieval | Age, ovarian stimulation protocol, BMI, physical activity, state and trait anxiety, infertility diagnosis, total energy intake and dietary supplements used (frequency and type of supplement) |
| Nassan et al., 2018 [32] | diet history questionnaires- intake of protein-rich foods | 171 | ICSI or conventional IVF | North America | 35 | 23.1 | high quartile patients for fish intake | low quartile patients for fish intake | During the previous year | Total daily calories, age, BMI, race, smoking status, daily supplemental dietary folate, supplemental vitamin B-12, iron intake, and supplemental omega 3 |
| Vujkovic, et al., 2010 [33] | diet history questionnaires | 107 | ICSI or conventional IVF.  Couples were excluded because of oocyte donation, or no oocytes could be retrieved, endometrioma, hydrosalpinx, medication error, or pregnancy before the start of the treatment | Netherlands | Not recorded | Not recorded | high adherence to a Mediterranean diet, eating more seafood, | low adherence to a Mediterranean diet, | During the 4 weeks before the trial | NR |

Abbreviations: NR- non-relevant. ICSI - Intracytoplasmic Sperm Injection

##### Table A.3: Studies included in the calculation of the odds ratio effect of omega-3 intake on pregnancy rates among patients who did not undergo fertility treatment: Features and patient characteristics

| **Study** | **Study design** | **Total (N)** | **Patient characteristics** | **Country** | **Mean age, y/ range** | **Mean BMI/ range** | **Type of Omega-3** | **Type of Control** | **Duration** | **Adjustment** |
| --- | --- | --- | --- | --- | --- | --- | --- | --- | --- | --- |
| Stanhiser et al., 2019 [35] | (Cohort study)  Non-random | 900 | Trying to conceive for fewer than 3 months and no history of infertility | USA | 30-44 | Not record-ed | Omega-3 supplement | Not taking omega-3 | Followed for up to 1 year until conception | Age, obesity, previous pregnancies, race, and vitamin D intake |
| Wise et al., 2018 [13] | (Cohort study)  Diet history questionnaires  (PRESTO)  Cohort study)  Diet history questionnaires  Snart Foraeldre (SF) | 653  553 | Had been trying to conceive for up to 6 menstrual cycles at the start of the study  Had been trying to conceive for up to 6 menstrual cycles at the start of the study | USA  Denemark | 29.8-30.6  28.3-28.0 |  | Higher quartiles of omega-3  Higher quartiles of omega-3 | Lowest quartile of omega-3  Lowest quartile of omega-3 | Followed for up to 12 months or until conception  Followed for up to 12 months or until conception |  |
| Gaskins et al., 2018 [36] | Diet history questionnaires | 501 | Who were planning pregnancy | USA | 18-44 | < 30 | Couples who consumed 8 or more seafood servings per menstrual cycle | Couples consuming 1 or less seafood servings | Followed for up to 1 year or until conception | Female and male age, race, male exercise, alcohol intake and sexual intercourse frequency |

##### Table A.4: Studies included in the calculation of the odds ratio effect of omega-3 intake on fertilization rates: Features and patient characteristics

| **Study** | **Study design** | **Total (N)** | **Patient characteristics** | **Country** | **Mean/ range Age, y** | **Mean/ range BMI** | **Type of Type of**  **Omega-3 Control** | | **Duration** | **Adjustment** |
| --- | --- | --- | --- | --- | --- | --- | --- | --- | --- | --- |
| Kim et al., 2010  [37] | Randomly divided into 2 groups | 900 | ICSI patients with a history of unexplained total fertilization failure | Korea | Not recorded | Not recorded | Omega-3 (1000 mg/day: 180 mg EPA + 120 mg DHA/day) | Group that did not take omega-3 for the duration of one menstrual cycle | One menstrual cycle | NR |
| Al-Alousi 2018  [29] | Randomized, double-blind, placebo-controlled clinical trial | 1290 | Subfertile ICSI patients.  No medical disorders or history of any diet in the previous three months. No tobacco and alcohol consumption, and supplementation of n-3 PUFAS in the past three months | Iraq | 20-40 | 18-34.99 | One 1000 mg omega-3 capsule daily | One daily placebo of 500 mg liquid paraffin | 8 weeks | NR |
| Nassan et al., 2018  [32] | Diet history questionnaires- intake of protein-rich foods | 171 | ICSI or conventional IVF | America | 35 | 23.1 | High quartile patients for fish intake | Low quartile patients for fish intake | During the previous year | Total daily calories, age, BMI, race, smoking status, daily supplemental dietary folate, supplemental vitamin B-12, iron intake, and supplemental omega 3 |

Abbreviations: NR- non-relevant. ICSI - Intracytoplasmic Sperm Injection.

##### Table A.5: Aggregated OR effect of omega-3 intake on clinical pregnancy rates in groups that underwent fertility treatment

| **Authors** | **Total (N)** | **Omega-3**  **intake**  **(N)** | **Control**  **(N)** | **Omega-3 intake** | | | **Control Omega-3** | | | | | | | | |  | | | | | | **OR** | | | | | | **95% CI of OR** | | | | | | | | |
| --- | --- | --- | --- | --- | --- | --- | --- | --- | --- | --- | --- | --- | --- | --- | --- | --- | --- | --- | --- | --- | --- | --- | --- | --- | --- | --- | --- | --- | --- | --- | --- | --- | --- | --- | --- | --- |
|  |  |  |  | **Success**  **Failure** | | | **Success Failure Type** | | | | | | | |  | | | | | |  | | | | | | **Lower** | | | | **Upper** | | | | |  |
| Bareksei et al., 2019 [28] | 1161 | 52 | 1109 | 36 | | 16 | 369 | | 740 | | | | Supplement | | | | | | 4.51 | | | | | | 2.47 | | | | 8.24 | | | |  |  |  |  |
| Al-Alousi, 2018 [29] | 115 | 59 | 56 | 20 | | 39 | 15 | | 41 | | Supplement | | | | | | 1.4 | | | | | | 0.32 | | | | | 1.58 | | | |  |  |  |  |  |
| Sugawa et al., 2018 [30] | 70 | 35 | 35 | 11 | | 24 | 12 | | 23 | | Diet history  questionnaire  (vegetables  and  seafood) | | | | | | 0.90 | | | | | | 0.30 | | | | | 2.69 | | | |  |  |  |  |  |
| Karayiannis et al., 2018 [31] | 165 | 86 | 79 | 43 | | 43 | 23 | | 56 | | Diet  Mediterranean  questionnaire | | | | | | 2.43 | | | | | | 1.2 | | | | | 6.25 | | | |  |  |  |  |  |
| Nassan et al., 2018 [32] | 171 | 88 | 83 | 45 | 43 | | 39 | 44 | | | | Diet | | | | | | 1.18 | | | | | | 0.64 | | | | 2.15 | | | | | |  |  |  |
| Vujkovic, et al., 2019 [33] | 107 | 53 | 54 |  |  | |  | | |  | | | | Diet  Mediterranean questionnaire | | | | | | 1.40 | | | | | | 1.01 | | | | 1.90 | | | | |  |  |

##### Table A.6: Aggregated OR effect of omega-3 intake on clinical pregnancy rates in groups that did not undergo fertility treatment

| **Authors** | **Total (N)** | **OR** | **95% CI of OR** | | **Omega-3** |
| --- | --- | --- | --- | --- | --- |
|  |  |  | **Lower** | **Upper** | **Type** |
| Stanhiser et al., 2019 [35] | 900 | 1.83 | 1.42 | 2.35 | Supplement |
| Wise et al.(PRESTO), 2018 [13]  Wise et al. Snart Fraeldre(SF), 2018 [13] | 653  553 | 1.40  0.96 | 1.13  0.78 | 1.73  1.19 | Diet  Diet |
| Gaskins et al., 2018 [36] | 501 | 1.44 | 1.04 | 2.01 | Diet |

##### Table A.7: Aggregated OR effect of omega-3 intake on fertilization rates in groups that underwent fertility treatment

| **Authors** | **Oocytes M2 Total(**inseminated or injected **(N)** | **Omega-3**  **intake**  **(N)** | **Control**  **(N)** | **Omega-3**  **intake** | | **Control** | | **Omega-3** | **OR** | **95% CI of OR** | |
| --- | --- | --- | --- | --- | --- | --- | --- | --- | --- | --- | --- |
|  |  |  |  | **Success** | **Failure** | **Success** | **Failure** | **Type** |  | **Lower** | **Upper** |
| Kim et al., 2010 [37] | 795 | 400 | 395 | 306 | 94 | 199 | 196 | Supplement | 3.20 | 2.37 | 4.34 |
| Al-Alousi 2018 [29] | 911 | 489 | 422 | 303 | 214 | 202 | 220 | Supplement | 1.77 | 1.19 | 1.99 |
|  |  |  |  |  |  |  |  |  |  |  |  |
| Nassan et al., 2018 [32] | 19 | 9 | 10 | 7 | 2 | 7 | 3 | Diet | 1.50 | 0.18 | 11.92 |
