# Supplementary material for: Effect of omega-3 supplements or diets on fertility in women: A meta-analysis
Source: Heliyon. 2024 Apr 6;10(8):e29324. doi: 10.1016/j.heliyon.2024.e29324 (PMC11019195; doi:10.1016/j.heliyon.2024.e29324)
Supplement: Multimedia component 2 [file mmc2.docx]

| Total quality score | Adequacy of follow-up of cohorts | Follow- up long enough for outcome to occurs | Assesment of outcome | Comparability of cohort | Outcome not present at start of study | Ascertainment of exposure | Selection of the non-exposed cohort | Representativeness of the exposed cohort | Study |
| --- | --- | --- | --- | --- | --- | --- | --- | --- | --- |
| 7 | * |  | * | ** | * | - | * | * | Sugawa et al., 2018 [30] |
| 7 | * |  | * | ** | * | - | * | * | Karayiannis et al., 2018 [31] |
| 8 | * | * | * | ** | * | - | * | * | Nassan et al., 2018 [32] |
| 6 | * |  | * | * | * | - | * | * | Vujkovic, et al., 2010 [33] |
| 7 | * | * | - | ** | * | - | * | * | Wise et al., 2018 [13]  (PRESTO) |
| 7 | * | * | - | ** | * | - | * | * | Wise et al., 2018 [13]  Snart Foraeldre (SF) |
| 7 | * | * | - | ** | * | - | * | * | Gaskins et al., 2018 [36] |
| 8 | * | * | * | ** | * | - | * | * | Stanhiser et al., 2019 [35] |

Table A.8: Risk of bias assessment of the included cohort studies according to the Newcastle-Ottawa Scale.
