# Supplementary material for: Effect of omega-3 supplements or diets on fertility in women: A meta-analysis
Source: Heliyon. 2024 Apr 6;10(8):e29324. doi: 10.1016/j.heliyon.2024.e29324 (PMC11019195; doi:10.1016/j.heliyon.2024.e29324)

**Figure A.1**. Assessments of the individual risk of bias in all RCT studies


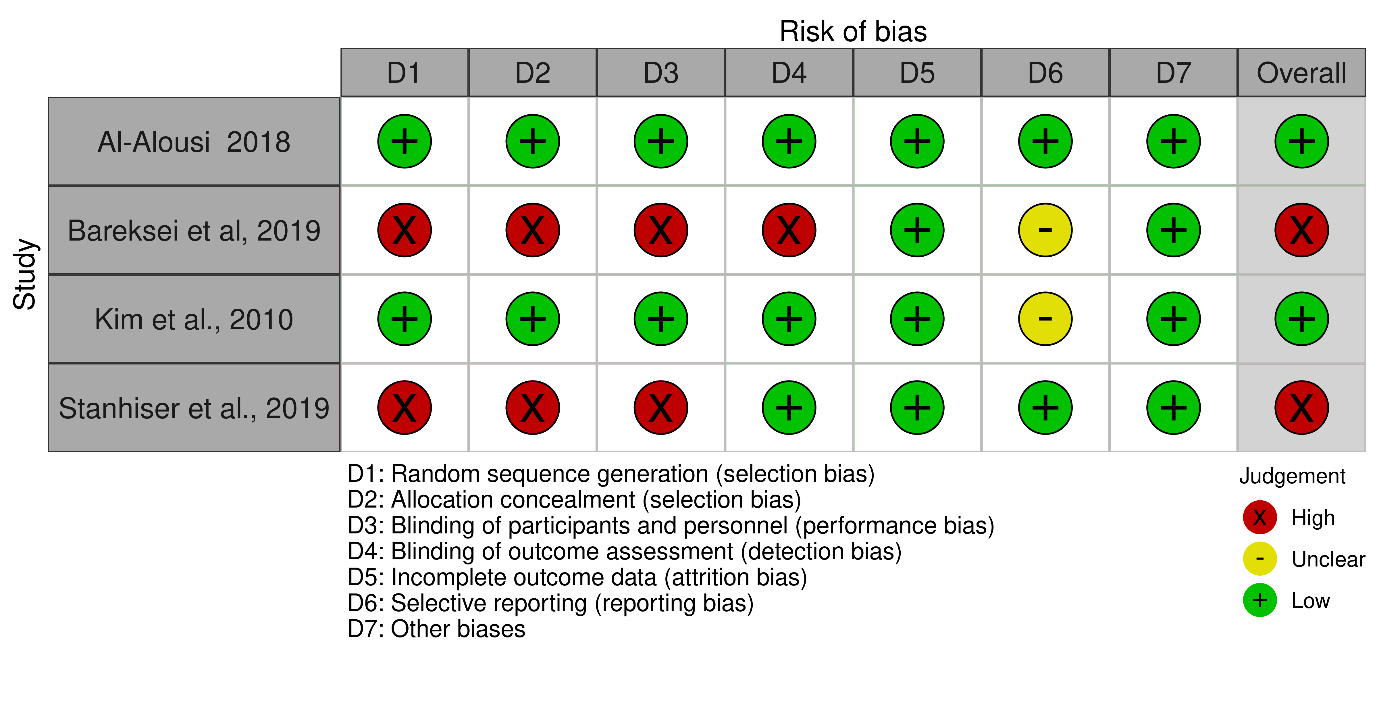


Low -There is no risk of bias in the study; Some concern – There is an intermediate level of bias risk in the study; High –There is a high risk of bias in the study.

**Figure A.2**. Summary plot of risk of bias in all RCT studies


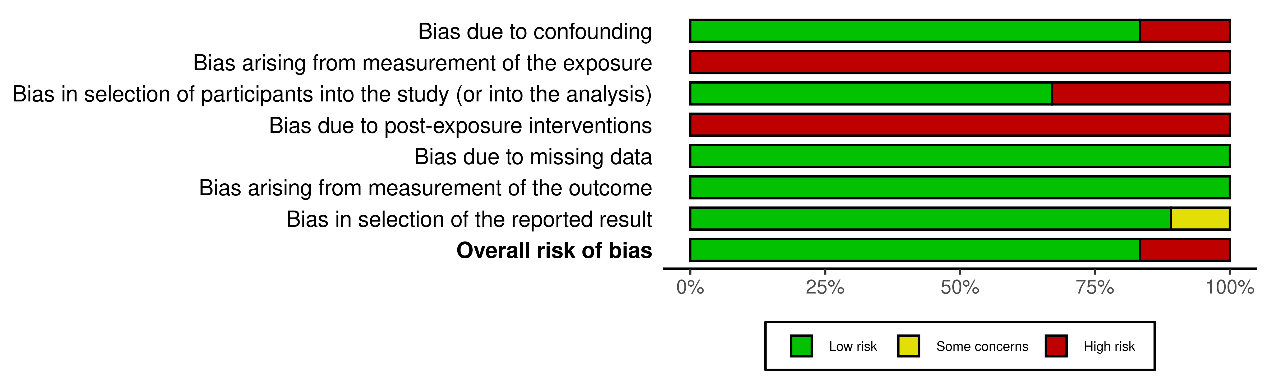

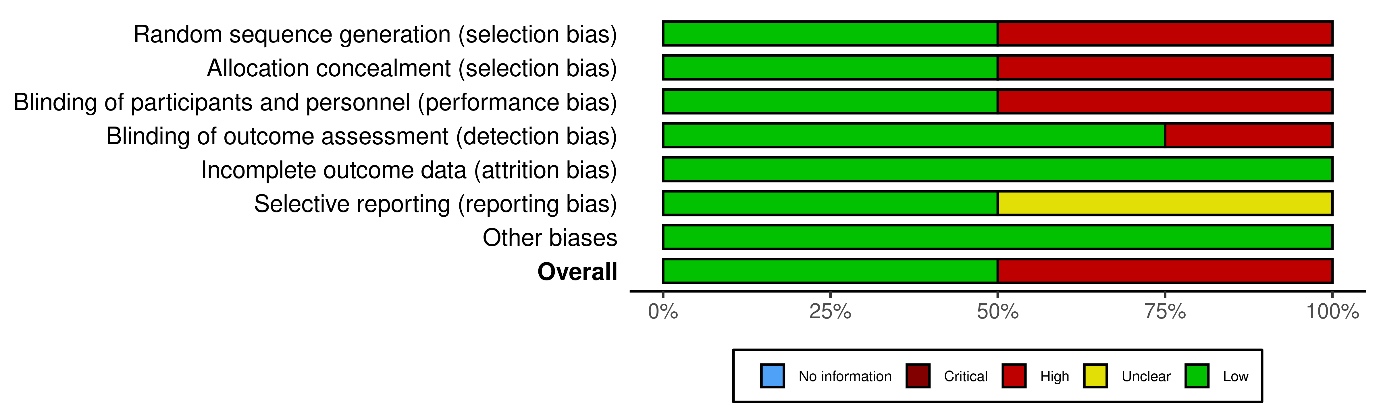


**Figure A.3**. Assessments of risk of bias in studies of omega-3 in the serum.


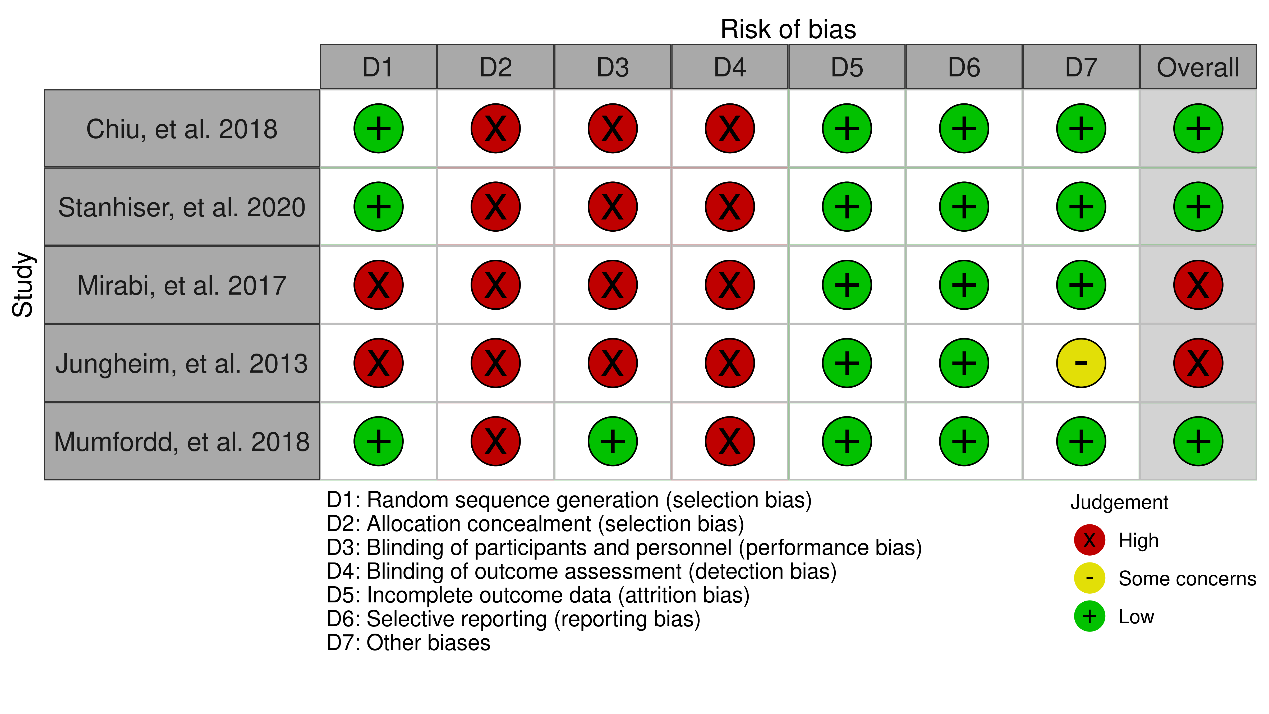


**Figure A.4**. Summary plot of risk of bias in studies of omega-3 in the serum.


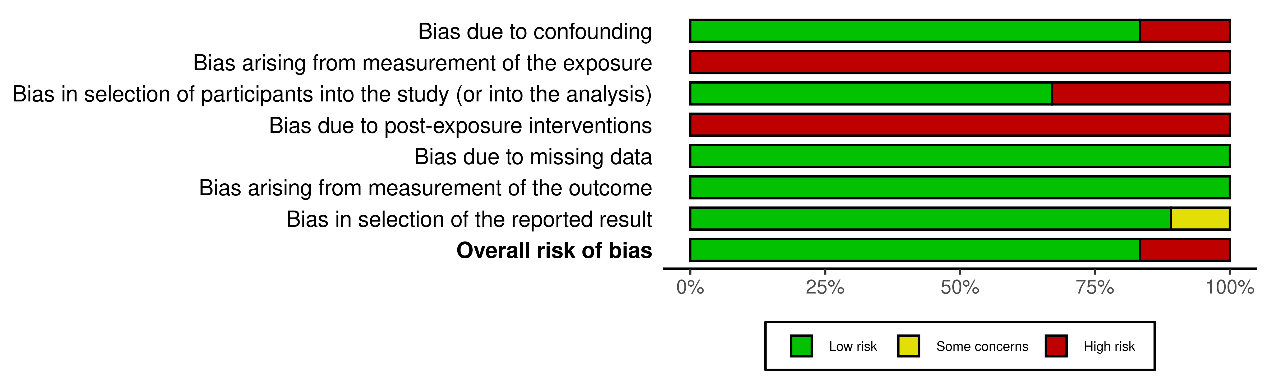

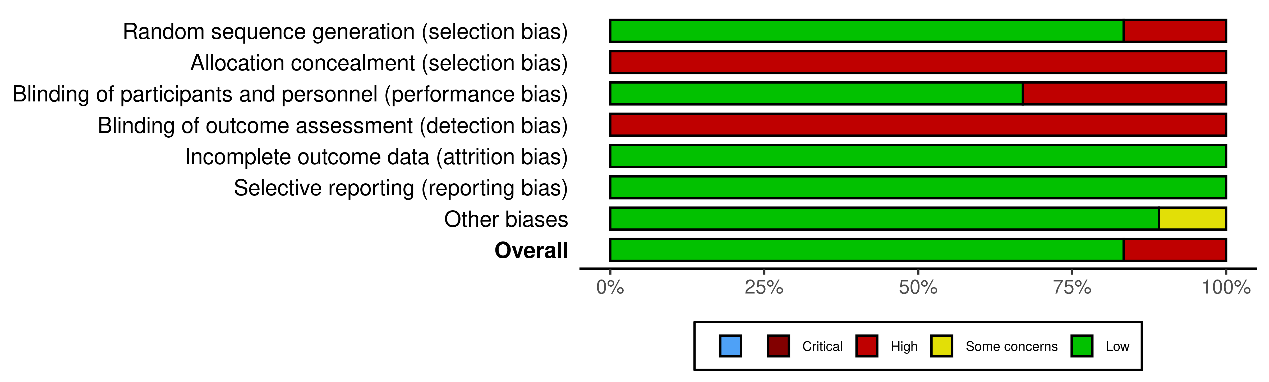

Supplement: Multimedia component 3 [file mmc3.docx]
